# Health-Related Physical Fitness as a Risk Factor for Falls in Elderly People Living in the Community: A Prospective Study in China

**DOI:** 10.3389/fpubh.2022.874993

**Published:** 2022-07-13

**Authors:** Hongxia Duan, Hao Wang, Yiwen Bai, Yan Lu, Xueping Xu, Jing Wu, Xubo Wu

**Affiliations:** ^1^Department of Rehabilitation, Shanghai Seventh People's Hospital Affiliated to Shanghai University of Traditional Chinese Medicine, Shanghai, China; ^2^School of Nursing, Shanghai Seventh People's Hospital, Shanghai University of Traditional Chinese Medicine, Shanghai, China; ^3^School of Rehabilitation Medicine, Shanghai Seventh People's Hospital, Shanghai University of Traditional Chinese Medicine, Shanghai, China

**Keywords:** LASSO, elderly, falls, community, prospective study, health-related physical fitness

## Abstract

**Objectives:**

Health-related physical-fitness (HRPF) involves multi-components of physical functional tests and is reported to be associated with the risk of fall. The study sought to determine whether specific physical fitness components were stronger predictors of falls among elderly people.

**Methods:**

This prospective cohort study involved 299 community residents age ≥60 years from Shanghai, China. The baseline data included comprehensive assessment of sociodemographic, clinical, and HRPF test. Subjects were followed for 1 year and were contacted by telephone to report falls. LASSO regression and Multivariate regression analysis were used to identify risk predictors of fall. In addition, we used receiver operating characteristic (ROC) curve analyses to determine whether the predictors have diagnostic.

**Results:**

During the 1-year prospective fall assessment, 11.7% of these subjects experienced one or frequent falls. LASSO models revealed that age (=0.01) and 8-ft up-and-go test score (=0.06) were positively associated with falls, while activity-specific balance confidence (ABC; = −0.007) and 2-min step test score (= −0.005) were inversely related. The Area Under roc Curve (AUC) for a linear combination of age, ABC scale score, 2-min step test and 8-ft up-and-go test was 0.778 (95% confidence interval: 0. 700–0.857), which was superior to any of the variables taken alone.

**Conclusion:**

Age, activity-specific balance confidence and fitness abnormalities were determined to contribute to the incident of falls. The value of 2-min step test score, and 8-ft up-and-go test score were the key HRPF components in predicting falls among elderly people.

## Introduction

Falls are a major public-health concern and considered as the second-leading cause of death from accidental injuries. Every year, an estimated 684,000 fatal falls occur, especially among the elderly, who are at high risk of falling (1). The consequences in frail elderly patients are often severe, especially hip fractures, which have profound effects on health-related quality of life and activities of daily living (ADL) in both male and female (2). In addition, the physical, psychological, and social outcomes after an elderly person falls are known to be poor, and increased fear of falling (FOF) is an independent risk factor for a subsequent fall (3, 4). Efficient fall prevention strategies are as important as the treatment of fall injuries in community-living older adults. To improve fall prediction, the assessment and detection of relevant factors are needed.

Activity limitation, altered consciousness, health condition, and environmental hazards are the most consistently reported risk factors for falls (5, 6). For the community-dwelling elderly, management recommendations of falls are increasingly emphasizing physical fitness as an important treatment target (7, 8). Health-related physical fitness (HRPF) as a component of physical fitness, includes eight items of quick and objective physical function test (9). It is indicated that the progressive loss of HRPF lead to increased risk of fall and physical inactive (7). However, it is unclear which key measures of HRPF could efficiently benefit for the fall prevention of older adults.

Although most descriptive and retrospective studies positively support older adults at higher risk of falls with worse HRPF outcomes (10–13), prospective evidence more rigorous and scientific for the HRPF at predicting falls among community-dwelling elderly is less clear. The previous prospective studies used logistic-regression models, Pearson's correlation coefficient (PCC), or covariate-adjusted regression models to analyze the correlation between falls and HRPF (14–17). Traditional regression techniques have practical limitations in analyzing multicollinear variables, which can cause fluctuations in the regression results and poor model stability. The Least Absolute Shrinkage and Selection Operator (LASSO) is a data analysis method that generally be utilized for risk factors selection and help to reduce multicollinearity of regression models. By introducing a penalty term in the model, unimportant regression coefficients are compressed to zero, and higher model prediction accuracy and generalization ability can be obtained at the cost of a certain estimation deviation (18, 19).

Currently, numerous studies have demonstrated an association between HPRF and fall risk. In view of multi-components trait of HPRF, evidence supporting its effectiveness in fall prediction remains ambiguous. Therefore, we conducted this prospective cohort study to explore the relationship between HRPF and falls, and evaluate the prognostic value of HRPF for fall prediction in the elderly *via* LASSO logistic regression.

## Methods

### Study Design and Participants

We conducted a 12-month prospective cohort study at the Gaoqiao community in Shanghai, China. Community-dwelling adults over the age of 60 years were eligible for study if they were clear consciousness and had no communication disorders. Exclusion criteria were: (1) severe cardiopulmonary dysfunction, (2) musculoskeletal diseases, (3) neurological dysfunction such as sensory impairment or motor paralysis; and (4) cognitive or psychological impairment.

**Figure 1:**
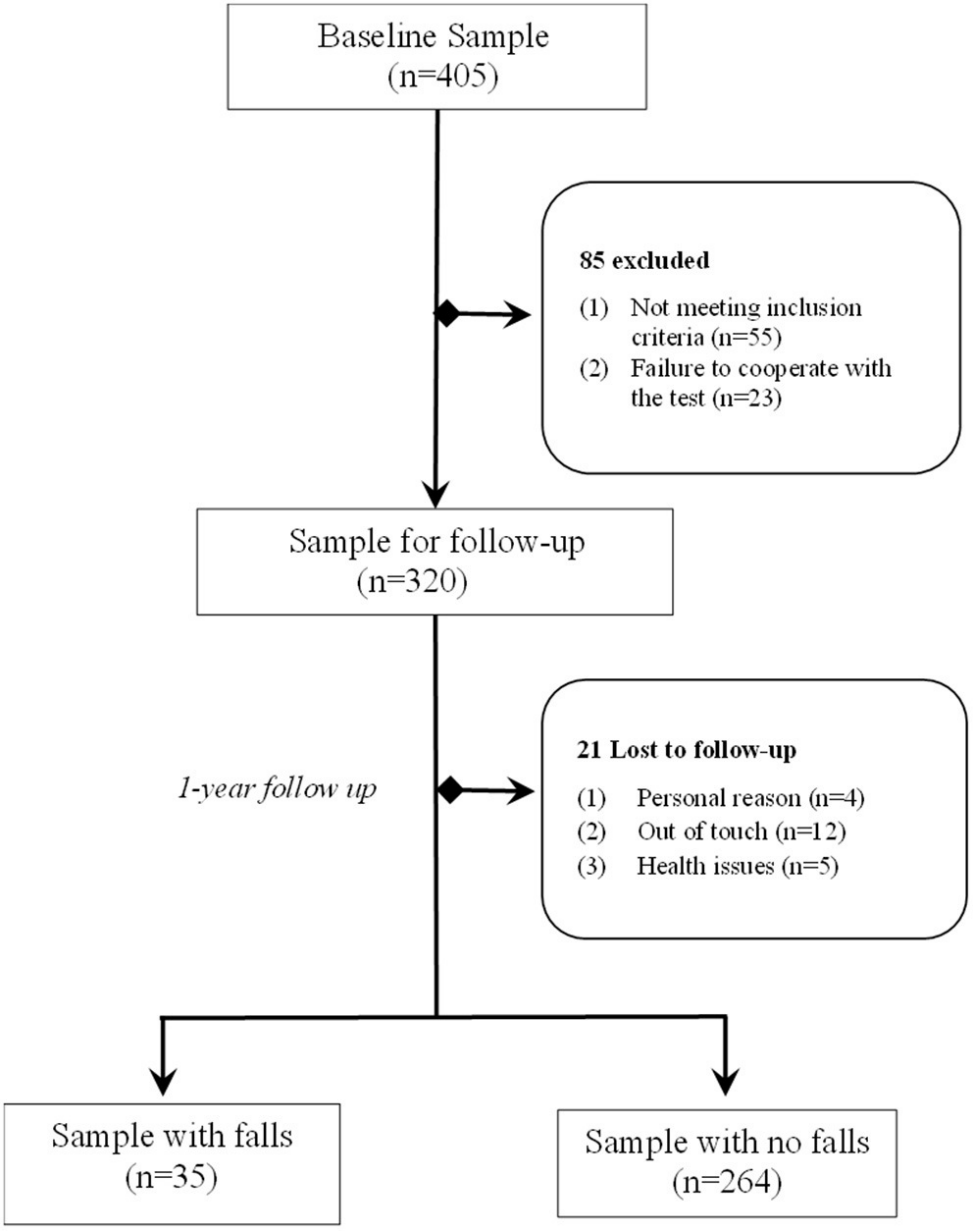
Flowchart of the study.

A baseline examination of 405 elders from June to September 2019 comprised of a physical examination, structured interview and HRPF test. Eighty-five subjects were excluded according to the exclusion criteria. The remaining 320 subjects were followed for 1 year and were contacted by telephone to specify the reported falls. Two hundred ninety-nine subjects (93.4%) completed the follow-up observation and were included in the present analysis. Detailed information of the study population is provided in Figure 1. The study was approved by the Ethics Committee at Shanghai Seventh People's Hospital (Number 2018-IRBQY-013) and was conducted in accordance with the Declaration of Helsinki.

### Measurements

#### Basic Characteristics

Demographic details including age; sex; BMI; smoking and drinking histories; and histories of hypertension, diabetes, stroke, and fractures were collected. We measured BMI using the InBody 720 body composition meter (InBody Co., Ltd, Seoul, Korea) and the bioresistance method. Before being measured, all subjects were instructed to have an empty stomach or to wait at least 2 h after a meal, to not refrain from urinating or defecating, and to not have performed moderate to high-intensity exercise within the previous 12 h.

#### Health-Related Physical Fitness

##### Activity-Specific Balance Confidence (ABC)

The ABC scale was designed to assess balance confidence in older adults. It asks participants to assess their confidence in keeping their balance without falling during 16 increasingly difficult activities, such as standing on a chair and walking up an icy sidewalk (20). Total score ranges from 0 to 100; the higher the score, the greater the participant's confidence in maintaining stability. Many studies have shown the ABC scale to have good reliability and utility as an independent predictor of falls in the community-dwelling elderly (20, 21).

##### Time “Up-and-Go” Test (TUGT)

This test is commonly used to examine functional mobility, balance, and fall risk. Subjects wear their regular footwear and use their customary walking aids (none, cane, or walker). With their walking aids at hand, they begin seated in a standard armchair (46-cm seat height) with their arms on the chair arms. They are told that at the word “start,” they are to stand up, walk 3 m away at a comfortable and safe pace, turn around, return to their chairs, and then sit down again. Elapsed time is recorded, and walking speed is calculated in ms (22).

##### Handgrip Strength (HGS)

This test is used as general muscle strength evaluation. We tested the subject's handgrip strength while seated in a stiff and stable straight-back chair with the subject's shoulder adducted and in a neutral position, the elbow flexed at 90 degrees, and the lower arm and wrist in a neutral position. The arm was not supported by a table, arm rest, or pillows. We showed the subject how to properly use the dynamometer before he performed the hand grip test. Subjects use the dominant hand to grip the grip dynamometer while seated. Over a period of 3 s, subjects were asked to increase their grip force from rest to maximal and maintain this force for 5 s. During measurement, the upper arm is kept close to the torso and the forearm perpendicular to the upper arm. Three measurements were taken, and the maximum value (in kg) was used (23).

##### 30-s Chair Stand (30-CS) Test

This test provides a valid and reliable measurement of a person's lower-limb muscle strength. The patient sits in a straight-backed armchair without leaning into the back, with feet shoulder width apart on the floor, arms crossed in front of the chest, and each hand on the opposite shoulder to ensure that no additional assistance is needed. After hearing the start instruction, the patient must rise completely and then return to the sitting position. The total number of attempts to complete this exercise within 30 s is recorded. If the patient has stood up when time is called, it is counted as one time (24).

##### 30-s Arm Curl (30-AC) Test

This test is used to measure upper-body endurance. Participants are seated in hard upright chairs with their backs straight and feet flat on the floor. The weight used for female is a 5-lb. (2.3-kg) dumbbell, for male an 8-lb. (3.6-kg) dumbbell. The weight is held in the dominant hand, perpendicular to the floor. Participants are asked to curl the weight by flexing their elbow while turning the palm of their hand toward their shoulder. At the “go” signal, participants should do as many elbow flexion movements as possible in 30 s (25).

##### 2-min Step Test

This test is used to measure aerobic endurance. While standing upright, the subject raises each knee to the midpoint of the connection between the patella and the anterior superior patella. The number of knee lifts within 2 min is recorded.

##### Sit-and-Reach (SR) Test

This test measures participants' flexibility. Subjects sit on a mat with both legs straightened out before them and feet flat against a test plate. Then, the arms are brought together and extended, the upper limbs are bent forward, and the middle finger and fingertips of both hands smoothly forward until it can no longer move.

##### Back Scratch (BS) Test

This test is used to assess upper-body flexibility. While standing upright, subjects stretch one hand down across their shoulders and extend the other hand from behind the waist. The distance between their two middle fingers is measured in cm.

##### 8-ft Up-and-Go Test

The result of this test is a dynamic indicator of functional performance, gait speed, and balance. Before the test, subjects stand behind the starting line. They are then asked to walk 2.4 m at their fastest possible speed in their usual footwear. Distance is marked using red tape on the floor. The digital stopwatch starts when the participant starts walking and stops when their forward foot crosses the finish line (26).

#### Definition of Fall

After HRPF measurements, individuals were followed up for about 1 year, during which they self-reported any falls. A fall was defined as an accident that causes a person to inadvertently lie on the floor or other lower level (1).

### Statistical Analysis

We conducted all statistical analyses using SPSS version 24.0 (IBM Corp., Armonk, NY, USA) and R software version 3.6.1 (R Foundation for Statistical Computing, Vienna, Austria). For continuous variables, normality data was expressed as mean ± standard deviation (SD), and a two-sample independent *t*-test was used for comparison between groups. Non-normally distributed data was represented by the median ± quartile interval, and the Mann–Whitney *U*-test was used for comparisons between groups. For count data, we used the chi-square test for intergroup comparisons.

**Figure 2:**
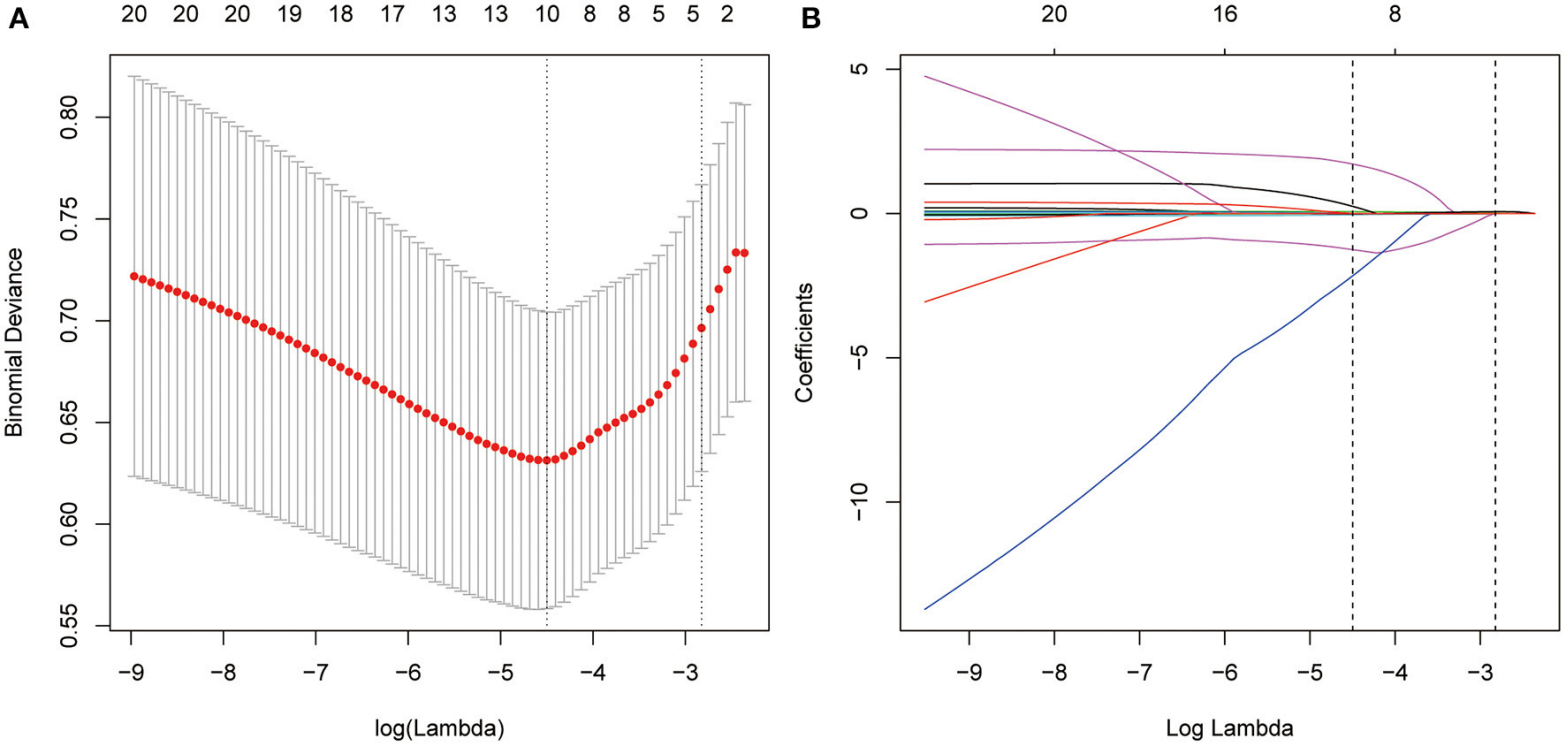
Texture feature selection using the least absolute shrinkage and selection operator (LASSO) binary logistic regression model. **(A)** Tuning parameter (λ) selection in the LASSO model used 10-fold cross-validation *via* minimum criteria. Dotted vertical lines were drawn at the optimal values by using the minimum criteria and the 1 standard error of the minimum criteria (the 1-SE criteria). A λ value of 0.0111, with log (λ),−4.4982 was chosen (1-SE criteria) according to 10-fold cross-validation. **(B)** LASSO coefficient profiles of the 12 texture features. A coefficient profile plot was produced against the log (λ) sequence. Vertical line was drawn at the value selected using 10-fold cross-validation, where optimal one resulted in 4 nonzero coefficients.

We used the “glmnet” package to perform the LASSO regression analysis. In order to fit the excellent model better, we evaluated the performance of LASSO regression using 10-fold cross-validation approach. R package will automatically produce two λ-one is the minimum binomial deviation, and the other is the maximum λ which is still within one standard error of the minimum binomial deviation. We choose the latter λ (λ = 0.0111; Figure 2) because it can give us a more parsimonious model. Finally, variables with non-zero regression coefficients were included in the final model. Furthermore, we drew a receiver operating characteristic (ROC) curve according to the variable screening results of LASSO regression to confirm the effectiveness of fall predictions. First step in multivariate receiver operating characteristic curve analysis was to run a logistic regression to save the predicted probabilities. Using this saved probability as an indicator, we then tested whether the Area Under the Curve (AUC) for the combined variables was significantly better than any of the variables alone. A value of *p*-value < 0.05 was considered statistically significant.

## Results

Of the 299 subjects, 29.10% were male and the average age was 67.16 ± 6.81 years. The most frequent comorbidity was hypertension (46.15%), followed by Diabetes (19.40%). During the 1-year follow-up, a total of 35 (11.7%) subjects had reported the incident of fall. Demographic details are shown in Table 1. The comparison between groups indicated that age was significantly higher in subjects with self-reported falls (*p* < 0.05). However, no significant difference was founded in gender, BMI, smoking and drinking history and disease history (all *p* > 0.05).

**Table 1:** Demographic details in participants with and without self-reported falls.

**Descriptive variable**	**Overall participants**	**Participants with self-reported falls**	**Participants without self-reported falls**	***p*-value**
	**(*n* = 299)**	**(*n* = 35)**	**(*n* = 264)**	
Mean age (years)	67.16 ± 6.81	71.77 ± 7.24	66.55 ± 6.53	<0.001
Sex (male, *n*, %)	87 (29.10)	11 (31.43)	76 (28.79)	0.747
BMI (kg/m^2^)	24.92 ± 3.06	25.44 ± 2.71	24.85 ± 3.10	0.287
Smoking history (*n*, %)	53(17.73)	4(11.43)	49 (18.56)	0.299
Drinking history (*n*, %)	48 (16.05)	5 (14.29)	43 (16.29)	0.452
Hypertension (*n*, %)	138 (46.15)	18 (51.43)	120 (45.45)	0.505
Diabetes (*n*, %)	58 (19.40)	7 (20.00)	51 (19.32)	0.924
Stroke (*n*, %)	18 (6.02)	3 (8.57)	15 (5.68)	0.346
Fracture history (*n*, %)	12 (4.01)	2 (5.71)	10 (3.79)	0.513

*BMI, body mass index*.

HRPF parameters of the two groups were presented in Table 2. In contrast, subjects with fall incident were more likely to have lower ABC score and poorer results of HRPF, including TUGT, 30-AC test, 30-CS test, 2-min step test, SR test and 8-ft up-and-go test (all *p* < 0.05). These results indicated that the fallers might have worse functional walking ability and limb endurance and strength. In addition, the comparison of HGS score and BS test score showed no statistical significance between groups (*p* > 0.05).

**Table 2:** HRPF indicators in participants with and without self-reported falls.

**Descriptive variable**	**Overall participants**	**Participants with self-reported falls**	**Participants without self-reported falls**	***p*-value**
	**(*n* = 299)**	**(*n* = 35)**	**(*n* = 264)**	
ABC (scores)	85.59 ± 12.89	76.26 ± 16.86	86.83 ± 11.76	<0.001
TUGT (m/s)	0.68 ± 0.16	0.59 ± 0.17	0.70 ± 0.15	<0.001
HGS (kg)	27.05 ± 8.17	25.00 ± 7.05	27.32 ± 8.28	0.114
30-AC test (times)	18.34 ± 5.17	14.86 ± 5.74	18.80 ± 4.92	<0.001
30-CS test (times)	20.86 ± 4.86	18.23 ± 4.12	21.21 ± 4.85	<0.001
2-min step test (times)	92.51 ± 15.08	87.54 ± 13.86	93.17 ± 15.14	0.038
SR test (cm)	6.73 ± 9.06	2.49 ± 10.18	7.29 ± 8.77	0.003
BS test (cm)	−10.58 ± 12.33	−13.97 ± 12.86	−10.13 ± 12.22	0.083
8-ft up-and-go test (s)	7.17 ± 2.40	9.32 ± 4.24	6.88 ± 1.87	<0.001

*ABC, activity-specific balance confidence; TUGT, time “up-and-go” test; HGS, handgrip strength; 30-AC, 30-s arm curl; 30-CS, 30-s chair stand; SR, sit-and-reach; BS, back scratch*.

In Table 3, LASSO logistic regression results showed that the four variables, age, ABC, 2-min step, and 8-ft up-and-go remained in the model and contributed to self-reported falls. Specifically, age (β = 0.01) and 8-ft up-and-go test (β = 0.06) were positively associated with falls, while ABC (β = −0.007) and 2-min step (β = −0.005) were inversely related to falls. In the model, the intercept was −2.25.

**Table 3:** The estimated coefficients for LASSO regression between sex, age, BMI, and HRPF variables with self-reported falls.

**Variables**	**Coefficients**
(Intercept)	−2.25
Age (years)	0.01
ABC (scores)	−0.007
2-min step test (times)	−0.005
8-ft up-and-go test (s)	0.06

*ABC, activity-specific balance confidence*.

Figure 2 shows the results of the 13 variables included in the LASSO regression and the corresponding λ coefficients of various penalty parameters. As λ = 0.0111, only four variables were retained in the model, which might have the greatest impact on self-reported falls. Specifically, age, ABC, 2-min step, and 8-ft up-and-go tests were the largest signal in the model.

The results of the multivariate logistic regression analysis further confirmed that increasing age (odds ratio [OR] = 1.35, 95CI%: 1.19–3.07) and the level of 8-ft up-and-go test (OR = 1.12, 95CI%: 1.03–1.32) were significant risk factors for fall (*p* < 0.05; Table 4). In terms of the ABC scale score, subjects with each 100 points higher scores had a 11% lower risk of fall (OR = 0.89, 95CI%: 0.72–0.96). The increasing level of 2-min step test indicted better aerobic endurance. The risk of fall decreased with the increase in 2-min step test levels. The predictive model was: Logit (fall) = −3.61166 + 0.07004^*^age – 0.03686^*^ABC – 0.01178^*^2-min step test + 0.11151^*^8-ft up-and-go test.

**Table 4:** Logistic regression model for risk factors associated with fall.

**Variables**	**Univariate analysis**			**Multivariate analysis**		
	**OR**	**95%*CI***	***p*-value**	**OR**	**95%*CI***	***p*-value**
Age (per 10 years)	1.56	1.27, 2.11	<0.001	1.35	1.19, 3.07	0.014
ABC /100 (scores)	0.81	0.70, 0.93	<0.001	0.89	0.72, 0.96	0.012
2-min step test (per 10 times)	0.78	0.63, 0.94	0.014	0.85	0.75, 0.98	0.033
8-ft up-and-go test (s)	1.32	1.17, 1.51	<0.01	1.12	1.03, 1.32	0.047

*ABC, activity-specific balance confidence*.

**Figure 3:**
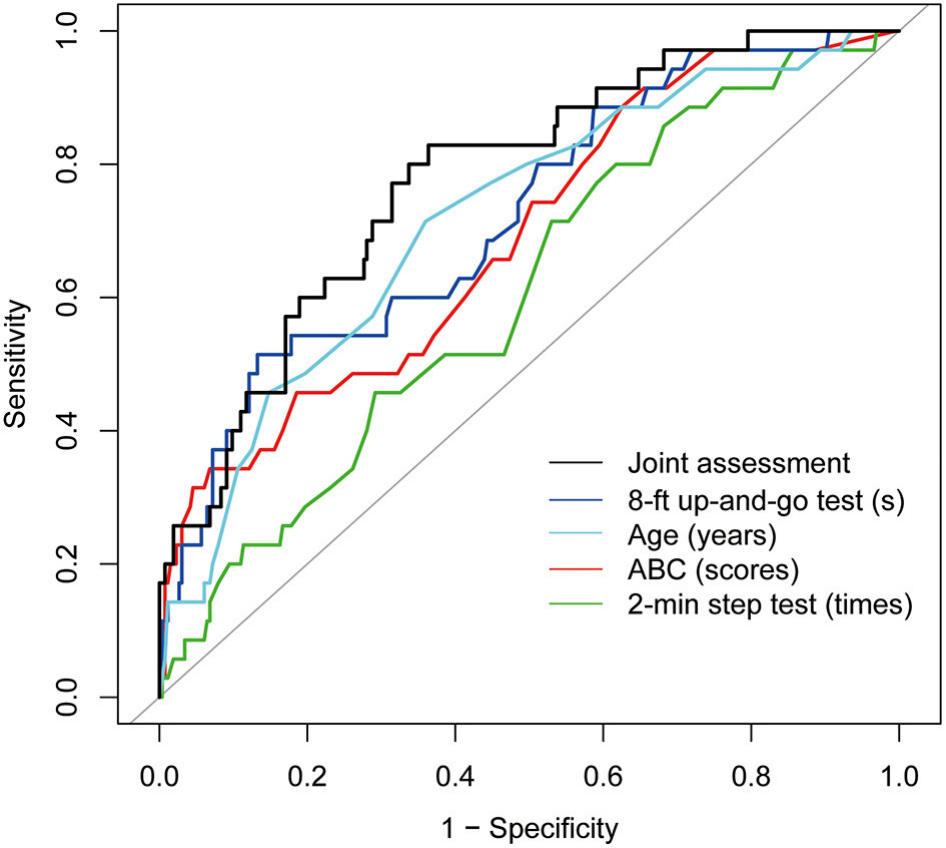
Colored lines represent receiver operating characteristic (ROC) curves of each variable for the prediction of fall events. 8-ft up-and-go test (AUC = 0.728, 95% confidence interval (CI): 0.636–0.820), Age (AUC = 0.718, 95% CI: 0.628–0.808), ABC (AUC = 0.689, 95% CI: 0.595–0.784), 2-min step test (AUC = 0.632, 95% CI: 0.521–0.744). Black line represents ROC curves of LASSO regression with the above four variables for the prediction of fall events. AUC = 0.778, 95% CI: 0.700–0.857.

Each of these was tested using ROC curve analysis with all 299 subjects. As shown in Figure 3, the AUC of predictors were: 8-ft up-and-go test [AUC = 0.728, 95% confidence interval (CI): 0.636–0.820], Age (AUC = 0.718, 95% CI: 0.628–0.808), ABC (AUC = 0.689, 95% CI: 0.595–0.784), 2-min step test (AUC = 0.632, 95% CI: 0.521–0.744). For multivariate receiver operating characteristic curve analysis, we first conducted a logistic regression to save the predicted probabilities. By using this saved probability as an indicator, the linear combination of the above 4 risks have been tested whether the resulting AUC is better than any of the risks taken alone. As showed in Figure 3, the linear combination of the risks resulted in an AUC of 0.778 (95% CI: 0.700–0.857, sensitivity was 0.829, and specificity was 0.636), which was superior to any of the variables taken individually.

## Discussion

In this study, we performed a HRPF-based screening of health and physical fitness status in 299 community-dwelling older adults. During the 1-year prospective fall assessment, 11.7% of these subjects experienced one or frequent falls. The incident of falls was associated with a lower walking speed and deteriorate lower limbs strength and agility as indicating by the baseline HRPF parameters. The results of LASSO and multi-regression models revealed that age, ABC scale score, specific HRPF components (including 2-min step test and 8-ft up-and-go test) were the main predictors of falls in elderly community residents. A combined assessment of these indicators may contribute to an early detection of falls.

Falls and fall-related injuries are significant health issue in the elderly population. It is reported that about one-third of people aged 65 and older fall at least once a year (27). The elders with bone and joint diseases or impaired consciousness have higher incidence of falls (28–30). In this cohort, ~12% of subjects reported fall event during the 1-year observation. This rate is relatively low compared to other prospective studies (31, 32). The baseline assessment had excluded the older people who had impaired physical mobility and health-related inactivity. Therefore, the participants in this study were physically and socially active, maintaining average levels of capacities and independence. Even the eldest participants had performed and completed the HRPF test. This might explain some inconsistency with results of other studies.

By multiple regression analysis, we demonstrated that age was an independent risk factor for falls. This result is consistent with most of other researches (33). It is well established that aging is associated with functional inefficiency and physical inactivity (34). In particular, people age ≥65 years are five times more likely to fall than younger people (35). This can be attributed to the decline in physical fitness and in psychological and cognitive function as well as the increase in comorbidities caused by age (35–38). Balance confidence is also closely related to fall risk in the elderly (39). Moderate-quality evidence indicates that the ABC scale has sufficient relevance in measuring balance confidence (40). Studies have demonstrated that ABC scores are significantly lower in fallers than in non-fallers during the 3 months preceding assessment (41). Indeed, recent evidence-based studies have shown that elderly people who have experienced accidental falls can develop FOF (42, 43), which is significantly associated with falls during the previous month (OR = 2.29, 95CI%: 1.78–2.95) (44). In addition, compared with objective functional performance, balance confidence seems to be more critical to fall risk (45). Therefore, caregivers should pay attention to balance confidence when caring for the elderly in the community.

HRPF has been proposed as a major marker of health status. The measurements of HRPF involves cardiovascular endurance, muscular strength, flexibility and agility (46). As a multi-components health-related indicator, HRPF usually consists of a set of structured, repetitive physical functional tests. In the baseline assessment, we had observed a total of eight functional fitness tests (i.e., TUGT, HGS, 30-CS, 30-AC, 2-min step test, SR, BS, and 8-ft up-and-go test). Considering the significant multicollinearity among these variables, a LASSO regression model was used for further data analysis. The results showed that 2-min step test score and 8-ft up-and-go test score were independent predictors for the risk of falls. Two-minute step test is one of alternatives for measuring the level of aerobic endurance. Ho et al. (47) evaluated cutoff thresholds for the association between physical performance and fall risk in elderly residents the Taipei City community, and revealed that <92 steps on the 2-min step test should be considered to indicate low cardiorespiratory fitness and could be used to identify the main target group at risk of falling.

The 8-ft up-and-go test measures speed, agility and balance while moving, and is regarded as a modified version of the TUGT. TUGT is a commonly used screening tool to assist clinicians to identify patients with mobility dysfunctions. Some recent studies suggested that the 8-ft up-and-go test was more feasible for use in a home setting, and has been identified as a superior predictor of fall among older people (11). In rheumatoid arthritis patients, Wilkinson et al. (48) reported that the 8-ft up-and-go test is the most appropriate measure of objective physical function. In this study, we found that the 8-ft up-and-go test, instead of TUGT, were significant predictors for falls in the LASSO analysis. Based on these results, we suggest that 2-min step test and 8-ft up-and-go test should be used in preference to other HRPF measures, for the prediction of falls. Moreover, the AUC for a linear combination of age, ABC score, measures of 2-min step test and 8-ft up-and-go test was superior to any of the variables taken alone. This result indicated that a combined assessment of these indicators may contribute to an early prevention of fall among the elderly.

Some limitations of this study should be mentioned. First, all observational studies are susceptible to residual confounding, unmeasured confounding, and measurement error. Second, falls were self-reported, and, as a result, the outcomes may be influenced by recall bias. Besides, the inclusion of baseline variables in this study was limited, so the four risk factors for falls in the results have certain limitations. The baseline variables can be expanded for further study. Finally, this was a single-center analysis with a possible sample bias. In the future, more multi-center studies with larger sample sizes and more baseline variables are required in this regard.

## Conclusions

In conclusion, we showed that LASSO regression could be a feasible option for narrowing and interpreting the role of a variety of health-related physical fitness measures and their relationships to falls in the elderly. We established fall-related factors including age, ABC, 2-min step test score, and 8-ft up-and-go test score. The results of our shrinkage analyses also showed a potential role of lower-limb muscle strength in the occurrence of falls, but this still needs to be confirmed in other prospective studies. Using statistical models to identify elderly people at high risk of falls could enabler faster predictions and possible interventions, which could lead to more-accurate health care and improve patient prognosis.

## Data Availability Statement

The raw data supporting the conclusions of this article will be made available by the authors, without undue reservation.

## Ethics Statement

The studies involving human participants were reviewed and approved by the Ethics Committee at Shanghai Seventh People's Hospital. The patients/participants provided their written informed consent to participate in this study.

## Author Contributions

HD: writing-most of manuscript. HW: writing-part of the manuscript, data curation and processing. YB, YL, and XX: data collection and follow-up. JW: software, writing—review and editing, and supervision. XW: methodology, writing—review and editing, and supervision. All authors contributed to the article and approved the submitted version.

## Funding

This study were funded by the National Natural Science Foundation of China (No. 71904127), Shanghai Municipal Commission of Health and Family Planning (No. 202150007), and Shanghai Pudong New District's National Traditional Chinese Medicine Development Comprehensive Reform Pilot Zone Construction Project (PDZY-2019-0501).

## Conflict of Interest

The authors declare that the research was conducted in the absence of any commercial or financial relationships that could be construed as a potential conflict of interest.

## Publisher's Note

All claims expressed in this article are solely those of the authors and do not necessarily represent those of their affiliated organizations, or those of the publisher, the editors and the reviewers. Any product that may be evaluated in this article, or claim that may be made by its manufacturer, is not guaranteed or endorsed by the publisher.
